# 2,6-Diamino-4-oxo-3,4-dihydropyrim­idin-1-ium chloride dihydrate

**DOI:** 10.1107/S1600536810031557

**Published:** 2010-08-11

**Authors:** Nura Suleiman Gwaram, Hamid Khaledi, Hapipah Mohd Ali

**Affiliations:** a Department of Chemistry, University of Malaya, 50603 Kuala Lumpur, Malaysia

## Abstract

In the crystal structure of the title compound, C_4_H_7_N_4_O^+^·Cl^−^·2H_2_O, adjacent cations are connected to one another through N—H⋯O hydrogen bonds, forming infinite chains along the *b* axis. These chains are further hydrogen bonded to the chloride anions and water mol­ecules, resulting in a three-dimensional network. The pyrimidine rings of adjacent mol­ecules are arranged in an anti­parallel manner above each other with centroid–centroid distances of 3.435 (1) Å, indicative of π–π inter­actions.

## Related literature

For related structures, see: Wijaya *et al.* (2004 ▶); Muthiah *et al.* (2004 ▶).
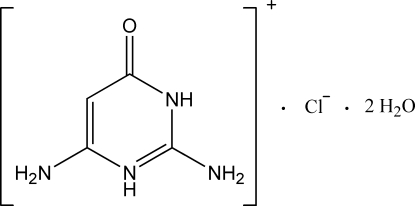


## Experimental

### 

#### Crystal data

C_4_H_7_N_4_O^+^·Cl^−^·2H_2_O
*M* *_r_* = 198.62
Monoclinic, 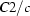

*a* = 20.4162 (4) Å
*b* = 6.6030 (1) Å
*c* = 12.8876 (2) Å
β = 107.903 (1)°
*V* = 1653.23 (5) Å^3^
*Z* = 8
Mo *K*α radiation
μ = 0.44 mm^−1^
*T* = 100 K
0.35 × 0.19 × 0.08 mm

#### Data collection

Bruker APEXII CCD diffractometer
Absorption correction: multi-scan (*SADABS*; Sheldrick, 1996 ▶) *T*_min_ = 0.862, *T*_max_ = 0.966
4405 measured reflections
1488 independent reflections
1352 reflections with (*I*) > 2.0σ(*I*)
*R*_int_ = 0.022

#### Refinement

*R*[*F* ^2^ > 2σ(*F* ^2^)] = 0.024
*wR*(*F* ^2^) = 0.066
*S* = 1.05
1488 reflections
139 parameters
10 restraints
H atoms treated by a mixture of independent and constrained refinement
Δρ_max_ = 0.22 e Å^−3^
Δρ_min_ = −0.25 e Å^−3^

### 

Data collection: *APEX2* (Bruker, 2007 ▶); cell refinement: *SAINT* (Bruker, 2007 ▶); data reduction: *SAINT*; program(s) used to solve structure: *SHELXS97* (Sheldrick, 2008 ▶); program(s) used to refine structure: *SHELXL97* (Sheldrick, 2008 ▶); molecular graphics: *X-SEED* (Barbour, 2001 ▶); software used to prepare material for publication: *SHELXL97* and *publCIF* (Westrip, 2010 ▶).

## Supplementary Material

Crystal structure: contains datablocks I, global. DOI: 10.1107/S1600536810031557/pv2317sup1.cif

Structure factors: contains datablocks I. DOI: 10.1107/S1600536810031557/pv2317Isup2.hkl

Additional supplementary materials:  crystallographic information; 3D view; checkCIF report

## Figures and Tables

**Table 1:** Hydrogen-bond geometry (Å, °)

*D*—H⋯*A*	*D*—H	H⋯*A*	*D*⋯*A*	*D*—H⋯*A*
O2—H8⋯O3	0.82 (2)	1.97 (2)	2.7503 (17)	161 (2)
O2—H9⋯Cl1^i^	0.81 (2)	2.51 (2)	3.2802 (11)	159 (2)
O3—H10⋯Cl1^ii^	0.83 (2)	2.39 (2)	3.2158 (12)	173 (2)
O3—H11⋯Cl1^iii^	0.84 (2)	2.35 (2)	3.1831 (13)	173 (2)
N4—H5⋯Cl1^iv^	0.85 (1)	2.45 (1)	3.2805 (13)	166 (2)
N4—H6⋯O1^i^	0.87 (1)	2.11 (2)	2.8310 (16)	141 (2)
N3—H4⋯O1^i^	0.87 (1)	1.88 (2)	2.6806 (15)	151 (2)
N2—H3⋯O2	0.88 (1)	2.05 (1)	2.9151 (17)	167 (2)
N2—H2⋯Cl1	0.87 (1)	2.38 (2)	3.2112 (13)	161 (2)
N1—H1⋯O2^v^	0.85 (1)	1.93 (1)	2.7727 (16)	174 (2)

Symmetry codes: (i) 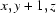; (ii) 
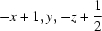
; (iii) 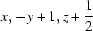; (iv) 
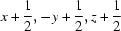
; (v) 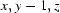.

**Fig. 1.**
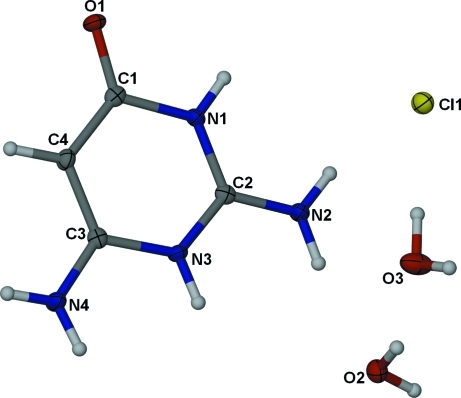
Thermal ellipsoid plot of the title compound at the 50% probability level. Hydrogen atoms are drawn as spheres of arbitrary radius.

**Fig. 2.**
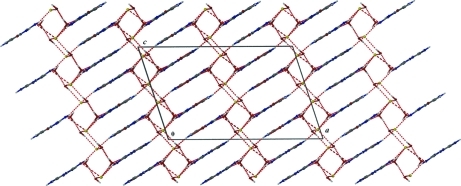
Packing view of the crystal structure, looking down the b-axis.

## supplementary crystallographic information

### Comment

The title compound is a chloride salt of 2,4-diamino-6-hydroxypyrimidine, cocrystallized with two molecules of water (Fig. 1). The structures of dimesylamide salt (Wijaya *et al.*, 2004) and sulfate salt (Muthiah *et al.*, 2004) of this cation have been reported previously. In the crystal structure of the title compound, adjacent diaminopyridinium cations are linked together *via* N—H···O hydrogen bonding into infinite chains along the *b*-axis. The pyrimidine rings of the adjacent molecules (related by symmetry: –x+3/2, -*y* + 1/2, -*z* + 1) are arranged in an antiparallel manner above each other with centroid-centroid distance of 3.435 (1) Å, indicative of a *π-π* interactions. The cation chains are hydrogen bonded to chloride anions and water molecules to form a three-dimensional hydrogen bonded network, involving O—H···O, O—H···Cl, N—H···Cl and N—H···O type hydrogen bonds (Tab. 1 & Fig. 2).

### Experimental

The pale yellow crystals of the title compound were obtained by slow evaporation of an aqueous ethanol (50%) solution of 2,4-diamino-6-hydroxypyrimidine in the presence of a few drops of hydrochloric acid.

### Refinement

The C-bound hydrogen atom was placed in idealized location (C—H = 0.95 Å) and refined as riding on its parent carbon atom. The nitrogen- and oxygen-bound hydrogen atoms were located in a difference Fourier map and were refined with distance restraints of N—H 0.88 (2) and O—H 0.84 (2) Å. *U_iso_*(H) were set to 1.2–1.5 × *U_eq_* (parent atom).

### Crystal data

**Table:**

C_4_H_7_N_4_O^+^·Cl^−^·2H_2_O	*F*(000) = 832
*M**_r_* = 198.62	*D*_x_ = 1.596 Mg m^−^^3^
Monoclinic, *C*2/*c*	Mo *K*α radiation, λ = 0.71073 Å
Hall symbol: -C 2yc	Cell parameters from 2897 reflections
*a* = 20.4162 (4) Å	θ = 3.3–30.5°
*b* = 6.6030 (1) Å	µ = 0.44 mm^−^^1^
*c* = 12.8876 (2) Å	*T* = 100 K
β = 107.903 (1)°	Block, yellow
*V* = 1653.23 (5) Å^3^	0.35 × 0.19 × 0.08 mm
*Z* = 8	

### Data collection

**Table:**

Bruker APEXII CCD diffractometer	1488 independent reflections
Radiation source: fine-focus sealed tube	1352 reflections with (*I*) > 2.0σ(*I*)
graphite	*R*_int_ = 0.022
φ and ω scans	θ_max_ = 25.2°, θ_min_ = 2.1°
Absorption correction: multi-scan (*SADABS*; Sheldrick, 1996)	*h* = −24→24
*T*_min_ = 0.862, *T*_max_ = 0.966	*k* = −7→7
4405 measured reflections	*l* = −15→13

### Refinement

**Table:**

Refinement on *F*^2^	Primary atom site location: structure-invariant direct methods
Least-squares matrix: full	Secondary atom site location: difference Fourier map
*R*[*F*^2^ > 2σ(*F*^2^)] = 0.024	Hydrogen site location: inferred from neighbouring sites
*wR*(*F*^2^) = 0.066	H atoms treated by a mixture of independent and constrained refinement
*S* = 1.05	*w* = 1/[σ^2^(*F*_o_^2^) + (0.0327*P*)^2^ + 1.7645*P*] where *P* = (*F*_o_^2^ + 2*F*_c_^2^)/3
1488 reflections	(Δ/σ)_max_ = 0.001
139 parameters	Δρ_max_ = 0.22 e Å^−^^3^
10 restraints	Δρ_min_ = −0.25 e Å^−^^3^

### Special details

**Table:**

Geometry. All e.s.d.'s (except the e.s.d. in the dihedral angle between two l.s. planes) are estimated using the full covariance matrix. The cell e.s.d.'s are taken into account individually in the estimation of e.s.d.'s in distances, angles and torsion angles; correlations between e.s.d.'s in cell parameters are only used when they are defined by crystal symmetry. An approximate (isotropic) treatment of cell e.s.d.'s is used for estimating e.s.d.'s involving l.s. planes.
Refinement. Refinement of *F*^2^ against ALL reflections. The weighted *R*-factor *wR* and goodness of fit *S* are based on *F*^2^, conventional *R*-factors *R* are based on *F*, with *F* set to zero for negative *F*^2^. The threshold expression of *F*^2^ > σ(*F*^2^) is used only for calculating *R*-factors(gt) *etc*. and is not relevant to the choice of reflections for refinement. *R*-factors based on *F*^2^ are statistically about twice as large as those based on *F*, and *R*- factors based on ALL data will be even larger.

### Fractional atomic coordinates and isotropic or equivalent isotropic displacement parameters (Å^2^)

**Table:**

	*x*	*y*	*z*	*U*_iso_*/*U*_eq_	
Cl1	0.485357 (17)	0.19327 (5)	0.09794 (3)	0.01545 (13)	
O2	0.58753 (5)	0.85326 (16)	0.25118 (9)	0.0160 (2)	
H8	0.5728 (9)	0.823 (3)	0.3011 (14)	0.024*	
H9	0.5575 (8)	0.910 (3)	0.2042 (14)	0.024*	
O3	0.55365 (6)	0.66656 (18)	0.41849 (10)	0.0223 (3)	
H10	0.5424 (10)	0.545 (2)	0.4079 (16)	0.033*	
H11	0.5331 (10)	0.709 (3)	0.4617 (15)	0.033*	
O1	0.75583 (5)	−0.12955 (15)	0.37901 (8)	0.0148 (2)	
N1	0.68878 (6)	0.14677 (19)	0.31998 (10)	0.0116 (3)	
H1	0.6566 (8)	0.062 (2)	0.2953 (13)	0.014*	
N2	0.61850 (6)	0.4221 (2)	0.25253 (10)	0.0140 (3)	
H2	0.5846 (8)	0.340 (2)	0.2242 (14)	0.017*	
H3	0.6124 (8)	0.554 (2)	0.2445 (13)	0.017*	
N3	0.73174 (6)	0.47140 (18)	0.35528 (10)	0.0113 (3)	
H4	0.7246 (8)	0.601 (2)	0.3510 (13)	0.014*	
N4	0.84541 (6)	0.53639 (19)	0.44953 (10)	0.0145 (3)	
H5	0.8853 (8)	0.496 (3)	0.4852 (13)	0.017*	
H6	0.8359 (9)	0.665 (2)	0.4492 (14)	0.017*	
C1	0.75285 (7)	0.0589 (2)	0.37417 (11)	0.0118 (3)	
C2	0.67858 (7)	0.3467 (2)	0.30834 (11)	0.0111 (3)	
C3	0.79694 (7)	0.3985 (2)	0.40909 (11)	0.0113 (3)	
C4	0.80775 (7)	0.1923 (2)	0.41736 (12)	0.0125 (3)	
H7	0.8525	0.1407	0.4524	0.015*	

### Atomic displacement parameters (Å^2^)

**Table:**

	*U*^11^	*U*^22^	*U*^33^	*U*^12^	*U*^13^	*U*^23^
Cl1	0.0137 (2)	0.0141 (2)	0.0164 (2)	−0.00100 (13)	0.00153 (14)	−0.00102 (13)
O2	0.0125 (5)	0.0165 (6)	0.0180 (6)	−0.0001 (4)	0.0033 (4)	0.0016 (4)
O3	0.0300 (7)	0.0152 (6)	0.0253 (6)	−0.0055 (5)	0.0137 (5)	−0.0032 (5)
O1	0.0153 (5)	0.0082 (5)	0.0191 (6)	0.0004 (4)	0.0027 (4)	0.0006 (4)
N1	0.0094 (6)	0.0100 (6)	0.0137 (6)	−0.0021 (5)	0.0012 (5)	−0.0012 (5)
N2	0.0106 (6)	0.0100 (6)	0.0192 (7)	−0.0012 (5)	0.0016 (5)	−0.0003 (5)
N3	0.0117 (6)	0.0077 (6)	0.0134 (6)	0.0006 (5)	0.0025 (5)	0.0004 (5)
N4	0.0114 (6)	0.0099 (6)	0.0190 (7)	0.0000 (5)	−0.0003 (5)	−0.0007 (5)
C1	0.0143 (7)	0.0125 (7)	0.0095 (7)	0.0016 (6)	0.0049 (5)	0.0002 (6)
C2	0.0128 (7)	0.0119 (7)	0.0097 (7)	−0.0009 (5)	0.0050 (6)	−0.0007 (5)
C3	0.0117 (7)	0.0136 (7)	0.0087 (7)	−0.0003 (6)	0.0034 (5)	0.0001 (5)
C4	0.0107 (7)	0.0127 (7)	0.0128 (7)	0.0016 (6)	0.0014 (6)	0.0005 (6)

### Geometric parameters (Å, °)

**Table:**

O2—H8	0.815 (15)	N2—H3	0.882 (14)
O2—H9	0.809 (15)	N3—C2	1.3484 (18)
O3—H10	0.832 (16)	N3—C3	1.3845 (18)
O3—H11	0.841 (15)	N3—H4	0.870 (14)
O1—C1	1.2467 (18)	N4—C3	1.3279 (19)
N1—C2	1.3379 (19)	N4—H5	0.846 (14)
N1—C1	1.4048 (18)	N4—H6	0.868 (14)
N1—H1	0.846 (14)	C1—C4	1.399 (2)
N2—C2	1.3142 (18)	C3—C4	1.378 (2)
N2—H2	0.867 (14)	C4—H7	0.9500
			
H8—O2—H9	108.9 (19)	H5—N4—H6	119.3 (17)
H10—O3—H11	105.1 (19)	O1—C1—C4	125.98 (13)
C2—N1—C1	123.52 (12)	O1—C1—N1	117.43 (13)
C2—N1—H1	122.2 (11)	C4—C1—N1	116.59 (13)
C1—N1—H1	114.3 (11)	N2—C2—N1	121.48 (13)
C2—N2—H2	118.8 (11)	N2—C2—N3	120.10 (13)
C2—N2—H3	120.8 (11)	N1—C2—N3	118.43 (12)
H2—N2—H3	120.4 (16)	N4—C3—C4	124.39 (13)
C2—N3—C3	121.99 (12)	N4—C3—N3	116.34 (13)
C2—N3—H4	118.6 (11)	C4—C3—N3	119.27 (13)
C3—N3—H4	119.4 (11)	C3—C4—C1	120.08 (13)
C3—N4—H5	118.1 (12)	C3—C4—H7	120.0
C3—N4—H6	121.9 (12)	C1—C4—H7	120.0

### Hydrogen-bond geometry (Å, °)

**Table:**

*D*—H···*A*	*D*—H	H···*A*	*D*···*A*	*D*—H···*A*
O2—H8···O3	0.82 (2)	1.97 (2)	2.7503 (17)	161.(2)
O2—H9···Cl1^i^	0.81 (2)	2.51 (2)	3.2802 (11)	159.(2)
O3—H10···Cl1^ii^	0.83 (2)	2.39 (2)	3.2158 (12)	173.(2)
O3—H11···Cl1^iii^	0.84 (2)	2.35 (2)	3.1831 (13)	173.(2)
N4—H5···Cl1^iv^	0.85 (1)	2.45 (1)	3.2805 (13)	166.(2)
N4—H6···O1^i^	0.87 (1)	2.11 (2)	2.8310 (16)	141.(2)
N3—H4···O1^i^	0.87 (1)	1.88 (2)	2.6806 (15)	151.(2)
N2—H3···O2	0.88 (1)	2.05 (1)	2.9151 (17)	167.(2)
N2—H2···Cl1	0.87 (1)	2.38 (2)	3.2112 (13)	161.(2)
N1—H1···O2^v^	0.85 (1)	1.93 (1)	2.7727 (16)	174.(2)

Symmetry codes: (i) *x*, *y*+1, *z*; (ii) −*x*+1, *y*, −*z*+1/2; (iii) *x*, −*y*+1, *z*+1/2; (iv) *x*+1/2, −*y*+1/2, *z*+1/2; (v) *x*, *y*−1, *z*.
